# Repurposing Known Drugs as Covalent and Non-covalent Inhibitors of the SARS-CoV-2 Papain-Like Protease

**DOI:** 10.3389/fchem.2020.594009

**Published:** 2020-11-16

**Authors:** Pietro Delre, Fabiana Caporuscio, Michele Saviano, Giuseppe Felice Mangiatordi

**Affiliations:** ^1^Department of Chemistry, University of Bari “Aldo Moro”, Bari, Italy; ^2^National Research Council (CNR) – Institute of Crystallography, Bari, Italy; ^3^Department of Life Sciences, University of Modena and Reggio Emilia, Modena, Italy

**Keywords:** drug repurposing, SARS–CoV–2, papain-like cysteine protease, molecular docking, molecular interaction fingerprints

## Abstract

In the absence of an approved vaccine, developing effective severe acute respiratory syndrome coronavirus 2 (SARS-CoV-2) antivirals is essential to tackle the current pandemic health crisis due to the coronavirus disease 2019 (COVID-19) spread. As any traditional drug discovery program is a time-consuming and costly process requiring more than one decade to be completed, *in silico* repurposing of existing drugs is the preferred way for rapidly selecting promising clinical candidates. We present a virtual screening campaign to identify covalent and non-covalent inhibitors of the SARS-CoV-2 papain-like protease (PLpro) showing potential multitarget activities (i.e., a desirable polypharmacology profile) for the COVID-19 treatment. A dataset including 688 phase III and 1,702 phase IV clinical trial drugs was downloaded from ChEMBL (version 27.1) and docked to the recently released crystal structure of PLpro in complex with a covalently bound peptide inhibitor. The obtained results were analyzed by combining protein–ligand interaction fingerprint similarities, conventional docking scores, and MM-GBSA–binding free energies and allowed the identification of some interesting candidates for further *in vitro* testing. To the best of our knowledge, this study represents the first attempt to repurpose drugs for a covalent inhibition of PLpro and could pave the way for new therapeutic strategies against COVID-19.

## Introduction

In the last two decades, three zoonotic spillovers of a coronavirus to humans have caused major epidemics, namely, the severe acute respiratory syndrome (SARS) epidemic of 2003 (more than 8,000 human infections and about 800 deaths) (Lu et al., 2020), the Middle East respiratory syndrome (MERS) outbreak of 2012 (about 2,500 confirmed cases and 858 deaths) (Lu et al., 2020), and the current Coronavirus Disease 2019 (COVID-19) pandemic (more than 15,700,000 confirmed cases and 637,810 deaths to date (WHO, COVID-19 daily report of July 26, 2020), the latter being the most devastating one. Coronaviruses are enveloped, single-strand, positive-sense RNA viruses infecting vertebrates and causing respiratory, enteric, and systemic diseases. The causative agent of COVID-19 has been named severe acute respiratory syndrome coronavirus 2 (SARS-CoV-2) (Gorbalenya et al., 2020) and belongs to the *Sarbecovirus* subgenus of the *Betacoronavirus* genus, which in turn belongs to the Coronaviridae family (Wu et al., 2020). SARS-CoV-2 RNA genome is about 79% identical to that of the highly pathogenic SARS coronavirus (SARS-CoV), which belongs to the *Sarbecovirus* subgenus as well, and 50% identical to that of the more recently emerged MERS-CoV, a member of the *Merbecovirus* subgenus of the *Betacoronavirus* genus (Llanes et al., 2020; Lu et al., 2020).

The most common manifestation of SARS-CoV-2 infection is pneumonia flanked by dry cough, dyspnea, and fever. Other manifestations include, e.g., gastrointestinal symptoms, leukopenia, fatigue, and/or loss of taste and smell. In the most severe cases, respiratory failure may occur and needs to be treated in an intensive care unit through mechanical ventilation. Life-threatening outcomes are frequently associated with elderly patients with concomitant diseases such as hypertension and cardiovascular diseases, chronic obstructive pulmonary disease (COPD), or diabetes. Finally, neurological complications, acute respiratory distress syndrome (ARDS), coagulation dysfunction, septic shock, and multiple organ dysfunction may follow, unfortunately leading to death (Lupia et al., 2020; Prezioso et al., 2020). In particular, ARDS arises as a result of hyperinflammation that is triggered by the viral infection and causes lung tissue damage (Freeman and Swartz, 2020). Hyperinflammation is characterized by the activation of the innate immune response, including the so-called cytokine storm, i.e., an excessive or uncontrolled release of proinflammatory cytokines such as interferons, tumor necrosis factor α, interleukin 6 (IL-6), and IL-1β (Tisoncik et al., 2012).

SARS-CoV-2 genome contains 14 open reading frames encoding (i) the spike (S), envelope (E), membrane (M), and nucleocapsid (N) structural proteins; (ii) the replicase/transcriptase polyproteins, which self-cleave to form 16 non-structural proteins (NSP1–NSP16); and (iii) accessory proteins. Non-structural proteins assemble into the replicase–transcriptase complex and include the papain-like protease (NSP3, PLpro), the main protease (NSP5, Mpro), the NSP7–NSP8 primase complex, the primary RNA-dependent RNA polymerase (NSP12), the helicase–triphosphatase (NSP13), the exoribonuclease (NSP14), the endonuclease (NSP15), and the N7- and 2′O-methyltransferases (NSP10 and NSP16) (Gordon et al., 2020). As in the case of SARS-CoV, SARS-CoV-2 entry into human cells is driven by the interaction of the viral S glycoprotein with the angiotensin-converting enzyme II (ACE2) receptor, which is highly expressed in alveoli, heart, and brain, whereas MERS-CoV uses dipeptidyl peptidase 4 (DPP4) to enter the host cells (Llanes et al., 2020; Zhou et al., 2020). Moreover, SARS-CoV-2 interacts with many different human proteins expressed in lung tissue, including, e.g., innate immune signaling proteins, histone deacetylase 2, epigenetic readers such as bromodomain proteins, proteins of the translational machinery, etc. (Gordon et al., 2020). Therefore, drugs able to disrupt the SARS-CoV-2 interactome, as well as drugs targeting viral proteins, may represent a feasible strategy to treat COVID-19.

Neither antiviral drugs nor a vaccine has been approved so far for SARS-CoV, MERS-CoV, and SARS-CoV-2. Treatments for COVID-19 are daily experimented by clinicians, and several clinical trials are ongoing. In the early stages of viral infection, therapies with antivirals designed for other viruses showed some beneficial effects. They include remdesivir, an anti–Ebola virus agent targeting viral RNA transcription; HIV-1 protease inhibitors such as the combination of lopinavir and ritonavir; and ribavirin, a molecule targeting the RNA polymerase and protein synthesis of different RNA viruses. On the contrary, in the advanced stages of COVID-19, antivirals are replaced by immunomodulatory agents targeting the host immune response such as the IL-6 receptor inhibitors tocilizumab, sarilumab, and siltuximab that are able to contain the cytokine storm (Song et al., 2020). Considering that developing an effective vaccine or a specific SARS-CoV-2 antiviral agent starting from scratch may take years, repurposing of approved drugs seems to be the quickest and most straightforward way to limit the burden of COVID-19 (Pinzi et al., 2020; Singh et al., 2020; Yamamoto et al., 2020). In this scenario, *in silico* drug-design tools can aid in the selection of the most suitable candidates. Moreover, at this stage of COVID-19 drug discovery research, structure-based approaches, which do not require a dataset of known active ligands to build a predictive model, are to be preferred. Indeed, since February 2020 the Protein Data Bank has collected up to 282 apo or holo structures of SARS-CoV-2 targets (accessed on July 14) that, together with the structures of human proteins entangled by the virus or responsible for some of its pathogenic effects, can be used to prioritize drugs for COVID-19 therapy. In particular, drugs can be repurposed as inhibitors of SARS-CoV-2 proteins, inhibitors of host proteins such as those involved in the immune response, or disruptors of virus–host interactions.

In this study, we present a structure-based virtual screening (VS) campaign to potentially repurpose 688 phase III and 1,702 phase IV clinical trial drugs from ChEMBL (version 27.1) (Gaulton et al., 2017) as covalent or non-covalent inhibitors of PLpro. To this aim, the recently released crystal structure of PLpro in complex with a covalently bound peptide inhibitor (PDB ID 6WX4) (Rut et al., 2020) was used for the first time. PLpro is a cysteine protease, and its activity consists in (i) the recognition of the LXGG motif and the subsequent hydrolysis of the peptide bond on the carboxyl side of glycine in the P1 position that results in the release of the NSP1, NSP2, and NSP3 proteins; (ii) deubiquitination; and (iii) deISGylation, i.e., the removal of the ubiquitin-like protein interferon-induced gene 15 from host proteins. It is noteworthy that these latter two activities interfere with the innate immune response to viral infection (Rut et al., 2020).

**Figure 1:**
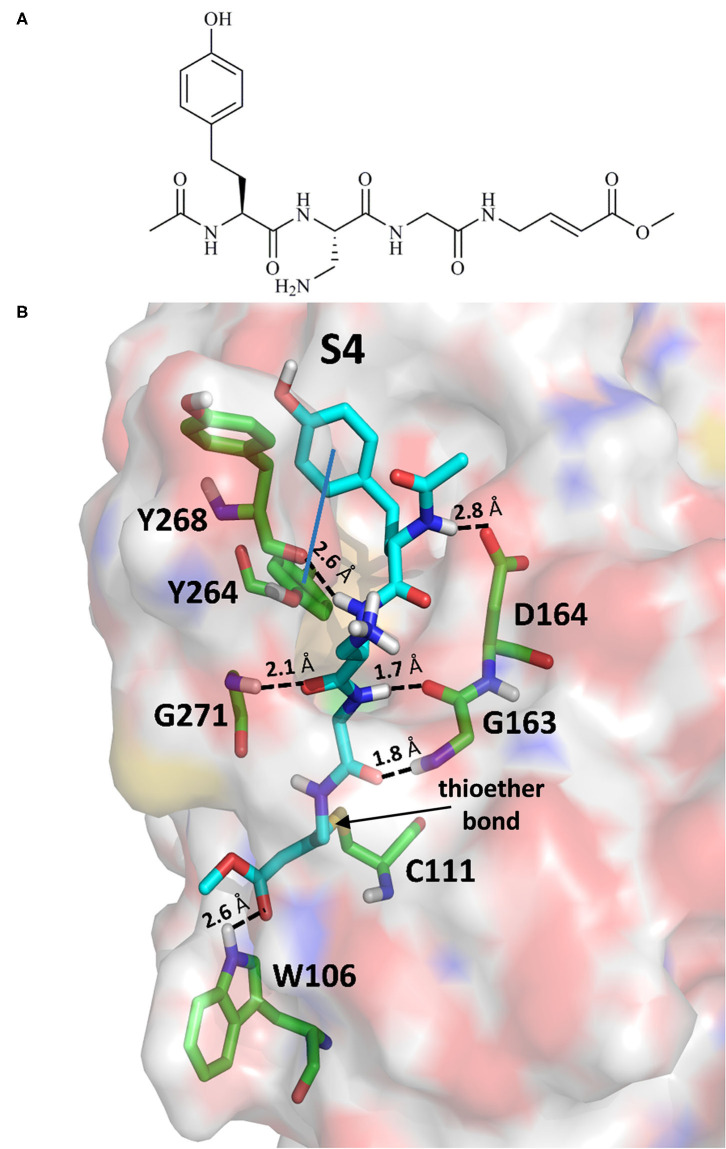
**(A)** 2D sketch of VIR251; **(B)** X-ray coordinates of VIR251 within the PLpro binding site (PDB ID 6WX4). VIR251 and important residues are rendered as sticks, whereas the protein is represented as a surface. H-bonds are represented by dotted black lines, whereas the pi-stacking interaction between VIR251 and Y264 is itemized by a blue line. For the sake of clarity, only polar hydrogen atoms are shown.

The 6WX4 cocrystallized inhibitor (VIR251; Figure 1A) is accommodated in the S4–S1 pockets of the catalytic site and makes a covalent bond with the catalytic C111, as a result of a Michael addition reaction that involves the β carbon of the vinyl group belonging to the VIR251 vinylmethyl ester moiety and the C111 thiol. Moreover, hydrogen bonds are formed with the backbone of G163, Y268, and G271 and with the side chains of W106, D164, and Y264, whereas inhibitor moieties at the P4 position are engaged in hydrophobic interactions (Figure 1B).

Therefore, a valuable candidate for drug repurposing should ideally mimic such interactions. Furthermore, other regions flanking the S4 pocket in the proximity of D164, Y273, and T301, not involved in accommodating VIR251, may be explored as well for the design of SARS-CoV-2 PLpro inhibitors (Rut et al., 2020).

## Materials and Methods

### Dataset Preparation

Candidate compounds were retrieved from ChEMBL (version 27.1), an open large-scale bioactivity database (Gaulton et al., 2017). In particular, all the compounds that reached phase III or phase IV clinical trials were selected. Subsequently, all duplicates were removed, and only molecules with a molecular weight (MW) in the range of 200 to 700 Da were retained. In this way, 2,390 chemicals were collected, including 688 phase III and 1,702 phase IV clinical trial drugs. All SMILES strings and compound names were extracted and collected in a smi file that was submitted to ligand preparation through the LigPrep tool, available from the Schrodinger Suite 2019-4 (LigPrep | Schrödinger, 2019), to build the 3D structures retaining the correct chirality specified in each SMILE string, desalt and generate all the tautomers and ionization states at a pH value of 7.0 ± 2.0 (LigPrep | Schrödinger, 2019).

### Non-covalent Docking Simulations

Molecular docking simulations were performed on the recently deposited X-ray structure of PLpro in complex with the peptide inhibitor VIR251 (PDB ID 6WX4, resolution: 1.66 Å) (Rut et al., 2020). The structure was preliminarily pretreated by using the Protein Preparation Wizard (PPW) tool (Protein Preparation Wizard | Schrödinger, 2019). More specifically, PPW added missing hydrogen atoms, reconstructed incomplete side chains, assigned the ionization states at physiological pH, set the orientation of any misoriented groups (N, Q, and H residues), removed water molecules farther than 3 Å from any atom of the cognate ligand, optimized the hydrogen bond network, and performed a restrained minimization using PPW default settings. Finally, before docking, all the water molecules were removed from the minimized protein structure (Madhavi Sastry et al., 2013). A cubic grid centered on the centroid of the VIR251 cognate ligand was generated, after breaking the bond between VIR251 and the C111 residue. An inner box of 10 Å × 10 Å × 10 Å and an automatic outerbox of 29 Å × 29 Å × 29 Å were built. Molecular docking simulations were carried out by using the Extra Precision (XP) protocol (Friesner et al., 2006). All docking simulations were performed using the default force field OPLS_2005, and during the docking process, the receptor protein was fixed, whereas full conformational flexibility was allowed for the ligands. Importantly, such a protocol was validated by redocking the cognate ligand VIR251 (RMSD = 0.79 Å).

### Covalent Docking Simulations

Covalent docking simulations were performed by using the covalent docking (CovDock, Maestro 12.2.012; Schrödinger LLC) workflow implemented in the Schrödinger suite 2019-4 (Zhu et al., 2014) and the previously pretreated 6WX4 crystal structure. A cubic grid, with an inner box and an automatic outer box having a side equal to 10 and 29 Å, respectively, was generated on the centroid of the VIR251 cognate ligand, C111 was selected as the reactive residue, and the Michael addition reaction was selected as the reaction type. Dataset compounds (total number equal to 2,390) were filtered in order to include only ligands matching the SMART pattern: [C,c]=[C,c]–[C,c,S,s]=[O]. Only 263 ligands potentially able to be engaged in a Michael addition reaction with C111 were retrieved and subsequently submitted to covalent docking simulations. Covalent docking consisted of five automatic steps (Zhu et al., 2014):

(i) for each molecule, conformations were generated by the ConfGen utility (Watts et al., 2010), and only the first three with the lowest conformational energies were submitted to a preliminary docking simulation in which the C111 residue was mutated to alanine to avoid steric clashes with the protein;
(ii) the C111 residue was restored, and docking poses in which the two atoms involved in the formation of the covalent bond are farther than 5 Å were discarded;
(iii) the covalent bond was then formed, and all changes in bond order, ionization state, or chirality were adjusted;
(iv) all covalent ligand–protein complexes were refined in order to restore standard bond lengths and avoid steric clashes. The obtained prime energy was used to rank the poses and select the most favorable binding geometry;
(v) finally, a docking score was assigned to the poses selected in the previous step. This score is defined as the average between the glide/docking score of the binding mode of the pre-reactive ligand and the glide/docking score of the ligand in the final covalent complex (Zhu et al., 2014).

CovDoc returned poses for 27 ligands that were ranked by docking score and analyzed by visual inspection. Importantly, such a protocol reproduced the binding mode of the VIR251 ligand (RMSD 1.5 Å).

### Protein–Ligand Interaction Fingerprints Generation

To generate the interaction fingerprints, a common binding site for all compounds was identified. In this regard, the ligand-binding site (BS) was defined using a cutoff radius of 6 Å from all the atoms of VIR251. Subsequently, the SIFt [Interaction Fingerprints (IFPs)] tool of Maestro (version 12.2.012, Schrödinger LLC) was applied to the selected docking poses, as well as to the VIR251 crystallographic coordinates, for computing the IFPs (Deng et al., 2004; Singh et al., 2006). Notably, the selected PLpro BS consists of 34 residues, each of which could potentially establish different chemical interactions with ligands. In particular, the presence of nine possible types of contacts have been verified: (i) any contact, (ii) backbone interactions, (iii) side-chain interactions, (iv) contacts with polar residues, (v) contacts with hydrophobic residues, (vi) formation of hydrogen bonds with H-bond acceptors of the BS, (vii) formation of hydrogen bonds with H-bond donors of the BS, (viii) contacts with aromatic residues, and (ix) contacts with charged residues. Therefore, each residue was represented by a nine-bit-long string for a total of 306 bits per string. A value equal to 1 means that an atom of the ligand is within the distance required to establish a specific interaction with a specific residue of the BS; on the contrary, a value equal to 0 indicates no contacts. The Tanimoto coefficient (TC) was used as a quantitative measure of the bit string similarity (Willett et al., 1998). The TC between two strings A and B is defined as follows:

$$\begin{array}{r} Tc=\frac{\left|A\;\cap B\right|}{\left|A\;\cup B\right|} \end{array}$$

where |*A* ∩ *B*| is the number of bits equal to 1 common to both *A* and *B*, and |*A* ∪ *B*| is the number of bits equal to 1 present in either *A* or *B*; the value of TC can range between 0 and 1, with 1 corresponding to two identical fingerprints. By selecting the VIR251 IFP as reference, TC was then calculated for all the docking poses generated in the previous step.

### MM-GBSA Calculations

Docking poses were submitted to a postdocking minimization using the MM-GBSA method (Genheden and Ryde, 2015), by allowing the flexibility of the residues at a maximum distance of 5 Å from the ligand. Default dielectric constants, the OPLS3 force field and the VSGB solvation model were used (Li et al., 2011). Prime MM-GBSA (Prime MM-GBSA | Schrödinger, 2019) outputs were ranked according to the Prime MM-GBSA ΔG (Bind) calculated as follows:

$$\begin{array}{r} \text{MM-GBSA }\Delta \text{Gbind}=\text{complex}-\text{ligand}-\text{receptor} \end{array}$$

where *complex* is the energy contribution calculated from the optimized ligand–receptor complex, and *ligand* and *receptor* are the energy contributions calculated from the optimized free ligand and free receptor, respectively. More negative values of ΔG (bind) indicate a stronger binding.

## Results and Discussion

### Candidates for Non-covalent PLpro Inhibition

A database of 1,702 approved drugs (i.e., currently in postmarketing surveillance trial) and 688 compounds that have reached phase III clinical trials was docked to the crystal structure of PLpro [PDB ID 6WX4 (Rut et al., 2020)]. Noteworthy, the high resolution (1.66 Å) and the presence of a cocrystallized peptide inhibitor make this PLpro structure, released on the May 20, 2020, particularly suitable for docking-based VS campaigns that to date have been only performed with PLpro homology models (Amin et al., 2020; Contreras-Puentes and Alvíz-Amador, 2020) or apo structures (Quimque et al., 2020). First, all the compounds were ranked according to their docking scores, and the 500 top-ranking molecules were kept for further evaluation. In particular, in order to overcome possible scoring function deficiencies (Marcou and Rognan, 2007), IFPs were computed for each ligand and compared to those obtained from the crystallographic coordinates of the peptide inhibitor VIR251 by computing a TC (hereinafter referred to as TC-IFP). Noteworthy, it has been shown that accounting for TC-IFPs in a VS campaign yields higher receiver operating characteristic curves and enrichments than ranking compounds based on the docking score only. In particular, compounds returning high TC-IFPs (i.e., ≥0.6) are more likely to be active (Marcou and Rognan, 2007) with respect to others with similar docking scores. Furthermore, all the top-500 compounds were submitted to MM-GBSA calculations in order to compute the binding free energies of the relative protein–ligand complexes. In order to properly estimate the energetic contribution of all the protein–ligand interactions, protein flexibility was incorporated during the calculations (see *Materials and Methods* for details). The selection of the most promising candidates was performed by considering the computed docking scores, TC-IFPs, and MM-GBSA–binding free energies. Furthermore, docking poses were carefully visually inspected in order to discard those with solvent-exposed hydrophobic groups or conformational artifacts. Special attention was given to the occupancy of regions in the proximity of the S4 pocket of the enzyme. As recently reported (Rut et al., 2020), regions flanking such a hydrophobic subcavity, although not involved in the VIR251 accommodation, are worth to be explored for designing PLpro inhibitors. Therefore, compounds protruding toward these regions were not discarded, albeit not fully mimicking the VIR251 binding mode reported in Figure 1B. Finally, a review of the available literature allowed us to privilege those compounds whose original therapeutic indication may be responsible for a desired polypharmacology to treat COVID-19 patients (Pinzi et al., 2020). Among the selected compounds, some inhibit the same protein (factor Xa) or the same family of proteins such as protein kinases (PKs) or viral/host proteases suggesting some similarity among the BSs. Moreover, other candidates belong to the same pharmacological class (e.g., antidiabetes, antihypertensives). Table 1 shows the 22 candidates selected for non-covalent PLpro inhibition along with their computed docking scores, TC-IFPs, and MM-GBSA–binding free energies.

**Table 1:** Candidate drugs for non-covalent PLpro inhibition.

**CHEMBL ID**	**Compound**	**2D structure**	**Docking score (rank)**	**TC-IFP (rank)**	**MM-GBSA score (rank)**	**Original mechanism of action**
**/**	**VIR251**		**−9.21**	**—**	**−91.27**	**PLpro inhibitor**
1421	Dasatinib	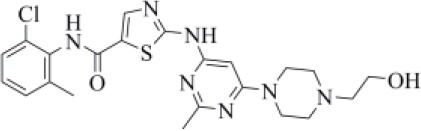	−10.46 (1)	0.648 (68)	−75.72 (71)	PK inhibitors
3813873	Pexidartinib	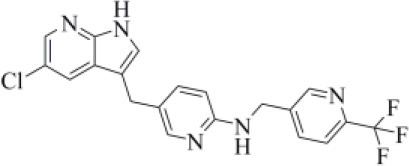	−6.24 (225)	0.582 (166)	−75.96 (68)	
3218576	Copanlisib	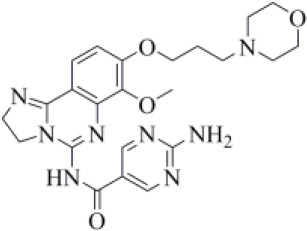	−6.90 (145)	0.494 (299)	−81.32 (40)	
116	Amprenavir	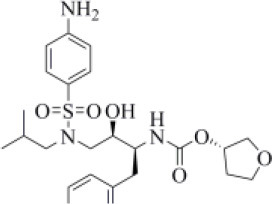	−5.50 (412)	0.677 (31)	−76.18 (67)	Protease inhibitors
115	Indinavir	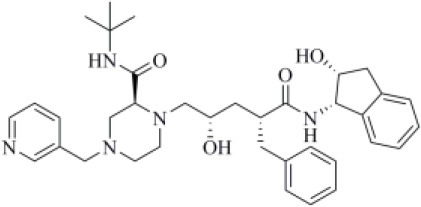	−6.31 (212)	0.513 (274)	−75.77 (70)	
1929396	Anagliptin	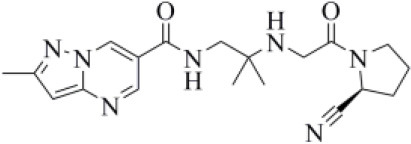	−6.08 (252)	0.687 (27)	−54.16 (258)	
218394	Boceprevir	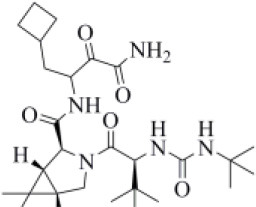	−6.01 (271)	0.507 (283)	−73.95 (83)	
520733	Semagacestat	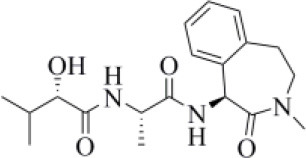	−9.36 (14)	0.591 (151)	−67.41 (128)	
1198857	Vilanterol	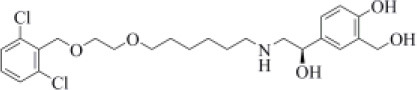	−5.78 (327)	0.653 (60)	−100.57 (1)	Adrenergic receptor modulators
1363	Arformoterol	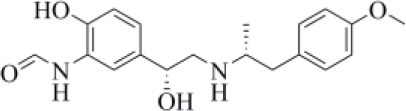	−5.61 (370)	0.662 (49)	−72.08 (93)	
24	Atenolol	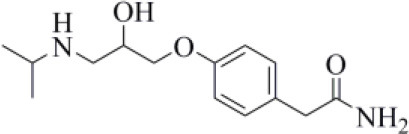	−5.47 (423)	0.613 (108)	−71.26 (97)	
515606	Cilazaprilat	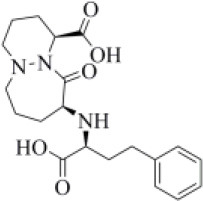	−5.79 (312)	0.682 (28)	−51.74 (275)	ACE inhibitors and direct oral anticoagulants
1269025	Edoxaban	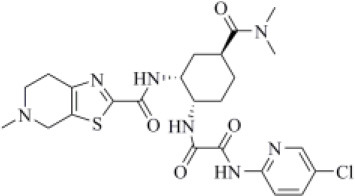	−7.97 (59)	0.762 (5)	−77.28 (61)	
198362	Rivaroxaban	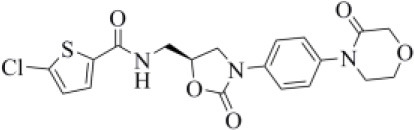	−5.26 (477)	0.587 (161)	−68.85 (115)	
2107723	Acotiamide	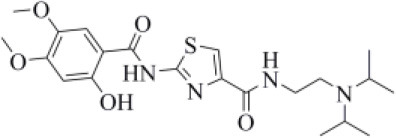	−8.07 (55)	0.706 (19)	−82.16 (35)	Drugs belonging to other classes
1200368	Bentiromide	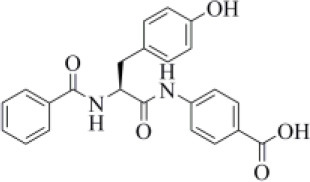	−8.80 (27)	0.781 (2)	−59.55 (200)	
2103929	Lymecycline	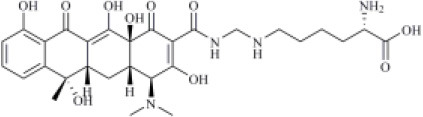	−6.78 (161)	0.701 (22)	−94.45 (8)	
2103841	Canagliflozin	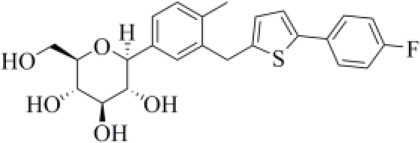	−5.87 (297)	0.530 (243)	−83.73 (28)	
4297185	Darolutamide	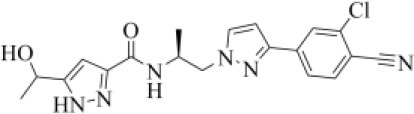	−10.03 (2)	0.698 (24)	−83.45 (30)	
1742461	Lafutidine	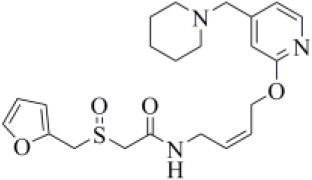	−8.20 (54)	0.662 (43)	−77.86 (57)	
439849	Vilazodone	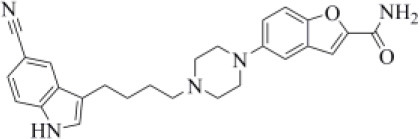	−5.98 (279)	0.742 (10)	−70.76 (101)	
34259	Methotrexate	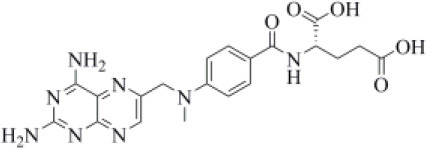	−5.41 (433)	0.458 (345)	−38.42 (408)	

*Docking scores and MM-GBSA–binding free energy values are reported in kcal/mol. The numbers in brackets indicate the corresponding rank positions. Docking and MM-GBSA scores obtained by redocking VIR251 (non-covalent docking protocol) are also reported*.

The presence, among the 22 selected compounds, of five inhibitors of other proteases (i.e., amprenavir, indinavir, anagliptin, boceprevir, and semagacestat) supports the reliability of the employed VS protocol. Importantly, amprenavir and indinavir have been already tested as inhibitors of SARS-CoV-2 replication returning EC_50_ values in the micromolar range (Yamamoto et al., 2020).

#### PK Inhibitors

**Figure 2:**
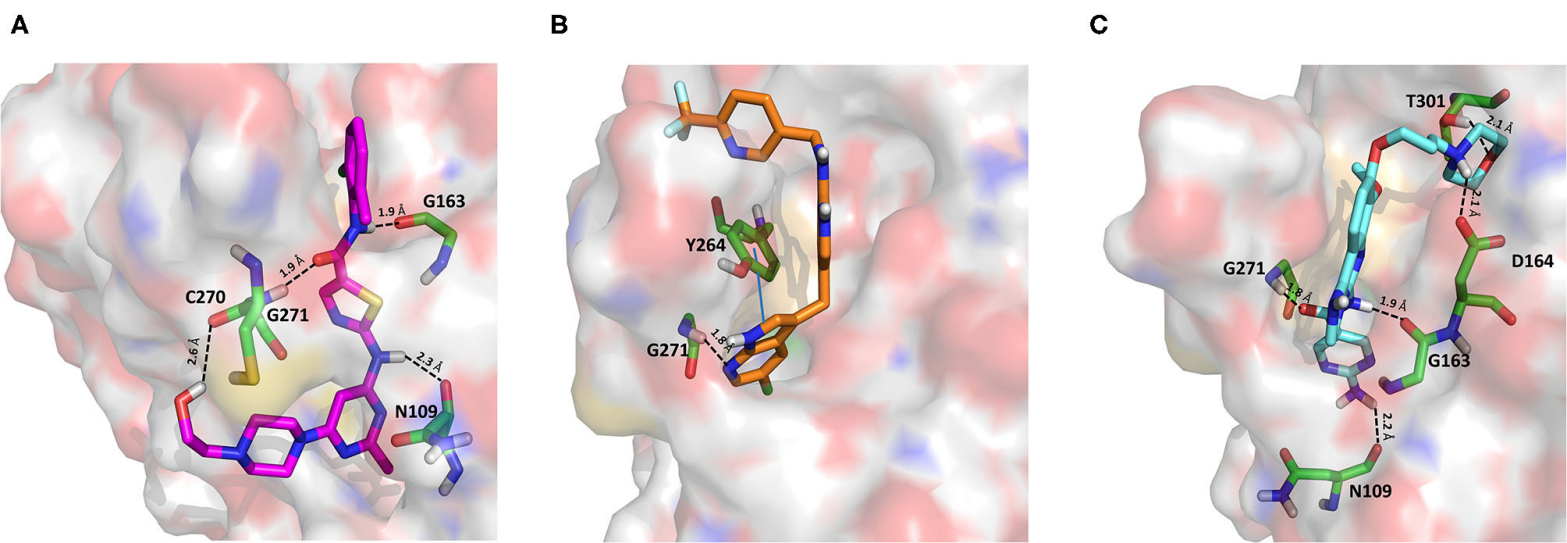
Top-scored docking poses of PK inhibitors selected for non-covalent PLpro inhibition: **(A)** dasatinib, **(B)** pexidartinib, and **(C)** copanlisib. Ligands and important residues are rendered as sticks, whereas the protein is represented as a surface. H-bonds are represented by dotted black lines, whereas the pi-stacking interaction between pexidartinib and Y264 is itemized by a blue line. For the sake of clarity, only polar hydrogen atoms are shown.

Dasatinib was approved by the Food and Drug Administration (FDA) about 15 years ago and is used for the treatment of chronic myelogenous leukemia (CML) and acute lymphoblastic leukemia (Keskin et al., 2016). It acts as an ATP-competitive inhibitor of different tyrosine kinases such as Bcr-Abl and the Src PK family (Keskin et al., 2016). The obtained data suggested that this drug could efficiently bind to PLpro. Among all the screened compounds, dasatinib returned the best docking score (−10.46 kcal/mol; Table 1), outperforming VIR251 (−9.213 kcal/mol). As confirmed by the computed TC-IFP (0.648), the predicted binding mode mimics that of VIR251. Dasatinib was predicted to establish four well-oriented H-bond interactions with the PLpro BS (Figure 2A), in particular with the G163 backbone C=O and G271 backbone NH (as observed for VIR251; Figure 1B), as well as with the backbone of N109 and C270.

Interestingly, in 2014, dasatinib showed promising antiviral activities against other coronaviruses such as MERS-CoV (EC_50_ = 17.6 μM) and SARS-CoV (EC_50_ = 2.1 μM) (Dyall et al., 2014) and has been recently used to treat a CML patient with a concomitant SARS-CoV-2 infection (Abruzzese et al., 2020). Therefore, it has been hypothesized that the ABL1 pathway could have an important role in viral replication. Our findings suggested an alternative explanation; i.e., the detected antiviral activity may be the result of PLpro inhibition.

Pexidartinib is a tyrosine kinase inhibitor recently approved by the FDA for the treatment of adults with symptomatic tenosynovial giant cell tumor (Benner et al., 2020). In particular, it works by inhibiting the colony-stimulating factor 1 receptor (Benner et al., 2020). Interestingly, pexidartinib returned an MM-GBSA–binding free energy comparable to that of dasatinib and a top-scored docking pose showing a binding mode in good agreement with that of VIR251 (TC-IFP = 0.582). In particular, as observed for the co-crystallized inhibitor, this compound interacts with the G271 backbone NH and the side chain of Y264, although by establishing a T-shaped pi-stacking rather than an H-bond interaction. It also shares with VIR251 the same orientation within the hydrophobic S4 pocket, as is evident by comparing Figures 1B, 2B. Noteworthy, pexidartinib is able to cross the blood–brain barrier (Butowski et al., 2016), a desirable property for treating patients because SARS-CoV-2 particles in the central nervous system (CNS) may be responsible for COVID-19 neurological manifestations (Baig et al., 2020; Zubair et al., 2020).

Copanlisib is a selective phosphoinositide 3-kinase (PI3K) inhibitor approved by the FDA for treating follicular lymphoma (Tarantelli et al., 2020). As reported in Table 1, it returned one of the best MM-GBSA scores (−81.32 kcal/mol). Copanlisib is able to mimic the binding mode observed for VIR251, in particular, by establishing H-bond interactions with the backbone of G163 and G271, as well as with the side chain of D164. An exception is represented by its morpholin group exploring a subpocket alternative to S4 where it is involved in an H-bond interaction with the T301 side chain. This evidence justifies, at least in part, the low TC-IFP value returned by this compound (0.482). Finally, an H-bond interaction with the N109 backbone C=O was also detected (Figure 2C). Noteworthy, Kindrachuk et al. showed that PK inhibitors targeting the PI3K/AKT/mTOR pathway are able to *in vitro* inhibit MERS-CoV replication, thus suggesting these compounds as promising tools for the treatment of coronavirus infections (Kindrachuk et al., 2015).

#### Protease Inhibitors

**Figure 3:**
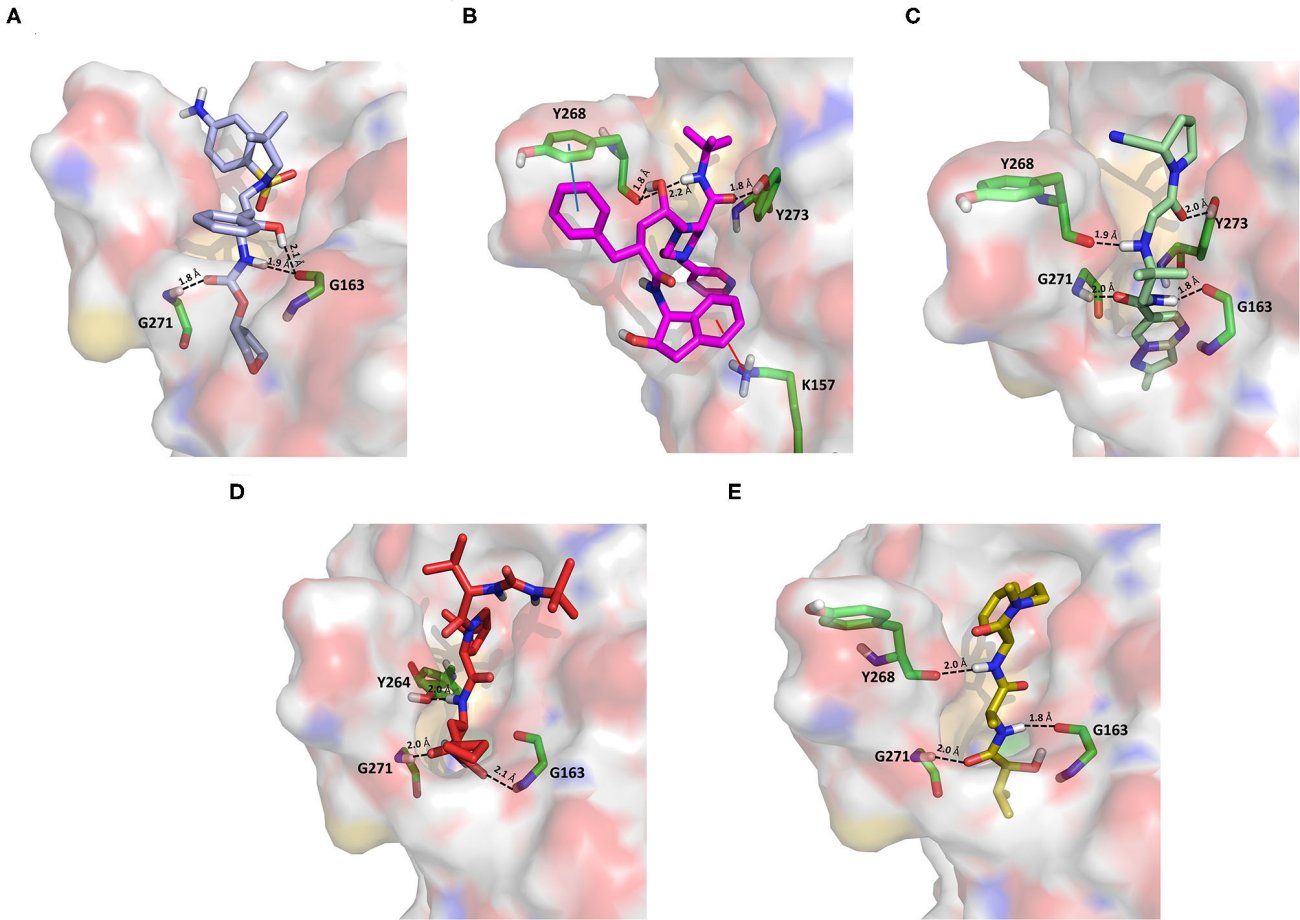
Top-scored docking poses of protease inhibitors selected for noncovalent PLpro inhibition: **(A)** amprenavir, **(B)** indinavir, **(C)** anagliptin; **(D)** boceprevir, and **(E)** semagacestat. Ligands and important residues are rendered as sticks, whereas the protein is represented as a surface. H-bonds are represented by dotted black lines, whereas the cation pi (pi-stacking) interaction between indinavir and K157 (Y268) is itemized by a red line. For the sake of clarity, only polar hydrogen atoms are shown.

Amprenavir is an HIV-1 protease inhibitor approved by the FDA in 1999 (Fung et al., 2000). Herein it was predicted as an efficient PLpro binder based on the computed MM-GBSA score (−76.18 kcal/mol). Notably, the obtained docking pose well-reproduces the binding mode of VIR251, as also shown by the high TC-IFP value (0.677). Amprenavir engages interactions with the backbone of both G271 and G163 and projects its P4 moiety in the S4 pocket as VIR251 (Figure 3A).

Noteworthy, this compound has been recently tested as a potential inhibitor of another SARS-CoV-2 protease (i.e., main protease, Mpro) and has been shown to be unable to inhibit Mpro at 20 μM (Ma et al., 2020). Finally, it has already been tested as an inhibitor of viral replication in SARS-CoV-2–infected VeroE6/TMPRSS2 cells (cells constitutively expressing the serine protease TMPRSS2, which confers a high susceptibility to SARS-CoV-2 infection) returning an EC_50_ value equal to 31.32 μM (Yamamoto et al., 2020).

Approved by the FDA in 1996 for the treatment of AIDS, indinavir is a selective HIV-1 protease inhibitor with good oral bioavailability (Plosker and Noble, 1999). Herein it was predicted to bind to the PLpro catalytic site with a high affinity (MM-GBSA score: −75.77 kcal/mol) by making two H-bonds with the backbone of Y268 and one with the side chain of Y273 (Figure 3B). In addition, a T-shaped pi-stacking interaction with Y268 and a cation-pi interaction with K157 were also detected. As amprenavir, indinavir has been recently proved to be an inhibitor of SARS-CoV-2 replication in cells (EC_50_ = 59.14 μM) (Yamamoto et al., 2020).

Anagliptin belongs to the class of “gliptins” (i.e., DPP4 inhibitors), which are antidiabetes drugs currently used by millions of patients and known to have a high safety profile (Nishio et al., 2015). It is still in phase III development in the United States and European Union, whereas in Japan it has been recently approved for use. Based on its docking pose, well-mimicking the VIR251 interaction pattern (TC-IFP: 0.687) and the predicted binding affinities, anagliptin may efficiently bind to PLpro. As shown in Figure 3C, this drug is able to make some interactions also established by the co-crystallized inhibitor, namely, the H-bonds with the backbone of G163, G271, and Y268. In addition, it forms also an H-bond with the side chain of Y273. It is worth to note that the administration of gliptins is expected to have beneficial effects on COVID-19 patients, with or without type 2 diabetes, because DPP4 is supposed to facilitate the entrance of SARS-CoV-2 in the airway tract (Solerte et al., 2020). These literature evidences, combined with the herein discussed results, put forward anagliptin as a drug that could modulate different relevant targets for COVID-19 therapy.

Boceprevir is an inhibitor of the non-structural protein 3/4A protease of the hepatitis C virus approved by the FDA in 2011 (Tungol et al., 2011). According to our results, boceprevir showed a favorable binding affinity to PLpro (MM-GBSA score: −73.95 kcal/mol). This finding is in agreement with a previous computational screening performed on a PLpro homology model (Elfiky and Ibrahim, 2020). Figure 3D shows the obtained top-scored docking pose. Boceprevir was predicted to establish H-bond interactions with the backbone of G163 and G271, as well as with the side chain of Y264, thus mimicking the binding mode of VIR251. This drug has recently been reported to be an inhibitor of SARS-CoV-2 Mpro (IC_50_: 4.13 μM) and a potent inhibitor of SARS-CoV-2 replication in cell culture (EC_50_: 1.31 μM) (Ma et al., 2020). Herein we hypothesize that its antiviral activity might be the result of a synergistic effect on the two SARS-CoV-2 proteases.

Semagacestat is a drug in phase III clinical trials for Alzheimer disease (AD) treatment and its activity is related to the inhibition of a multisubunit protease complex named γ-secretase (Doody et al., 2013). Interestingly, this compound outperformed VIR251 in terms of docking score (−9.36 vs. −9.21 kcal/mol) and returned a good MM-GBSA score (−67.41 kcal/mol), as well as a top-scored docking pose almost mimicking the binding mode of the cocrystallized inhibitor (TC-IFP: 0.591). In particular, as VIR251, semagacestat makes H-bonds with the G163 backbone C=O, the G271 backbone NH, and the Y268 side chain OH (Figure 3E). Being developed to treat AD, this compound is able to efficiently cross the blood–brain barrier, a required property to treat neurological manifestations in COVID-19 patients.

#### Adrenergic Receptor Modulators

**Figure 4:**
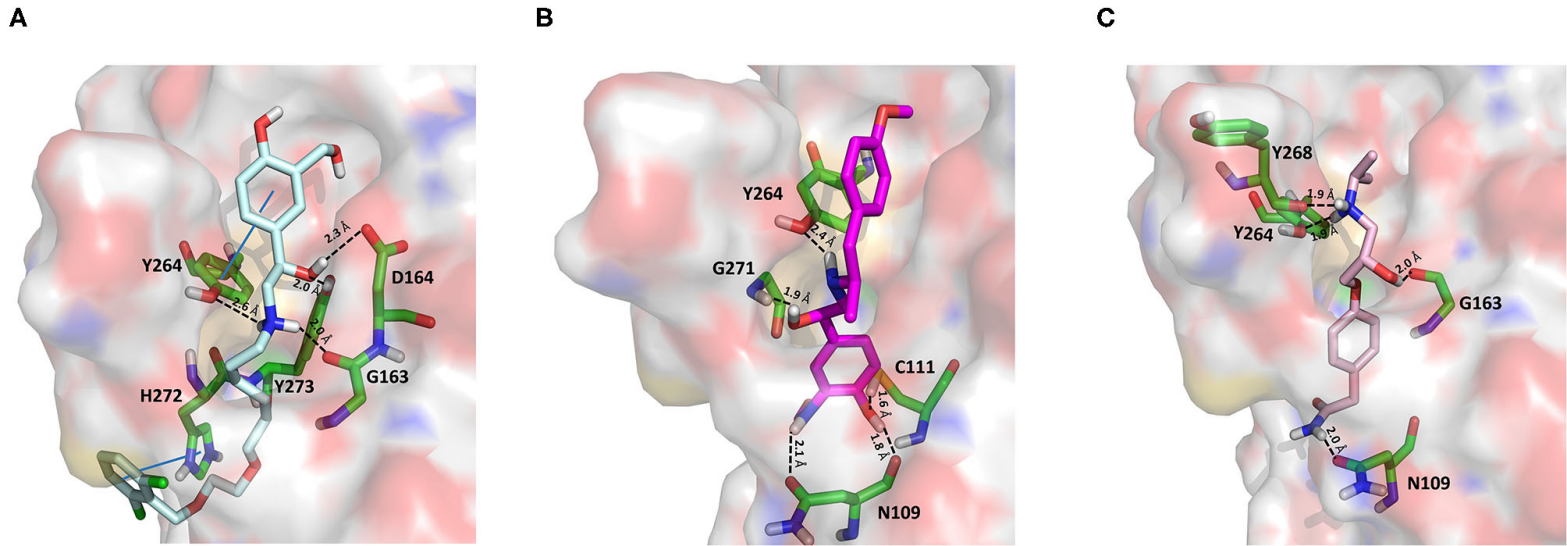
Top-scored docking poses of adrenergic receptor modulators selected for non-covalent PLpro inhibition: **(A)** vilanterol, **(B)** arformoterol, and **(C)** atenolol. Ligands and important residues are rendered as sticks, whereas the protein is represented as a surface. H-bonds are represented by dotted black lines, whereas the pi-stacking interaction between Vilanterol and H272 is itemized by a blue line. For the sake of clarity, only polar hydrogen atoms are shown.

As an agonist of the β2-adrenoreceptor, vilanterol was approved by the FDA in 2013 for the treatment of COPD (Ramadan et al., 2019). Herein, it was predicted to bind to PLpro with a high binding affinity as it returned the best MM-GBSA score (−100.57 kcal/mol) among all the screened compounds (Table 1), exceeding the predicted binding free energy of VIR251 (−91.27 kcal/mol). Importantly, its docking pose was predicted to be consistent with the binding mode of the cognate ligand (TC-IFP: 0.653), as shown in Figure 4A, making vilanterol all the important interactions for VIR251 recognition, such as H-bonds with the G163 backbone C=O and the side chains of D164 and Y264.

Additional interactions involve an H-bond with the side chain of Y273 and a T-shaped pi-stacking with H272 and Y264. As recently emphasized by Deslée et al. (2020), several evidences suggest a link between COVID-19 infection and COPD. More specifically, a higher expression of the ACE2 receptor has been observed in COPD patients. Therefore, vilanterol may show potential polypharmacological effects of interest for treating COVID-19 patients with COPD.

As vilanterol, arformeterol is a β2-adrenoreceptor agonist approved for COPD treatment (King, 2008). It showed a good predicted binding affinity to PLpro (MM-GBSA score: −72.08 kcal/mol) and a binding mode consistent with that of VIR251 (TC-IFP: 0.662). As reported in Figure 4B, arformeterol interacts with the G271 backbone NH and the side chain of Y264, as VIR251. Other H-bond interactions involve the side chain of C111, as well as the side chain and backbone of N109.

Atenolol is a second-generation cardioselective β1-adrenergic antagonist, approved by the FDA in 1981. This drug is widely used for the management of hypertension, angina pectoris, cardiac dysrhythmias, and myocardial infarction (Rehman et al., 2020). In particular, atenolol has reached, in the United States, more than 20 million prescriptions in 2017 The Top 300 of 2020. Although relatively small (MW = 266 Da) compared to VIR251 (MW = 480 Da), this compound returned a good MM-GBSA score (−71.26 kcal/mol) and TC-IFP (0.613). Interestingly, atenolol makes the majority of the interactions observed for VIR251, namely, H-bond interactions with the backbones of G163 and Y268, as well as with the side chain of Y264. In addition, a well-oriented H-bond with the side chain of N109 was also detected (Figure 4C). Noteworthy, the beneficial effect of β-adrenergic blockers for the treatment of COVID-19 patients has been recently hypothesized by Vasanthakumar on the basis of their ability to reduce “the mortality in respiratory failure, ARDS, and septic shock conditions” (Vasanthakumar, 2020).

#### ACE Inhibitors and Direct Oral Anticoagulants

**Figure 5:**
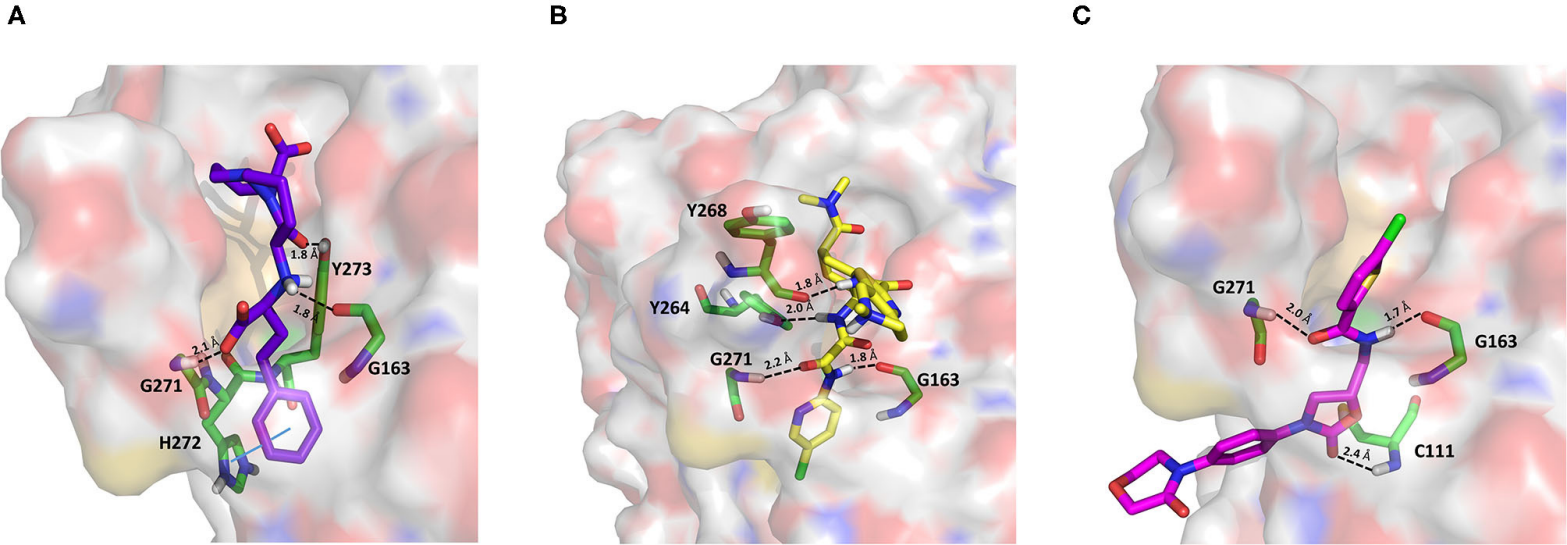
Top-scored docking poses of ACE-is and direct oral anticoagulants selected for non-covalent PLpro inhibition: **(A)** cilazaprilat, **(B)** edoxaban, and **(C)** rivoraxaban. Ligands and important residues are rendered as sticks, whereas the protein is represented as a surface. H-bonds are represented by dotted black lines, whereas the pi-stacking interaction between **Cilazaprilat** and H272 is itemized by a blue line. For the sake of clarity, only polar hydrogen atoms are shown.

Cilazapril is a prodrug and is converted by carboxylesterases to cilazaprilat, a member of the class of angiotensin-converting enzyme inhibitors (ACE-is), i.e., drugs blocking the conversion of angiotensin I to angiotensin II (Deget and Brogden, 1991). Because of their ability to reduce cytokine production, ACE-is have been proposed as a possible therapeutic intervention to decrease the intensity of the host response to SARS-CoV-2 infection. Even if some authors have suggested that the upregulation of ACE2 expression induced by a chronic use of ACE-is may be linked to the most severe outcomes associated with COVID-19, this hypothesis has not yet been experimentally confirmed. Moreover, some studies have shown lower IL-6 plasma levels, a lower rate of progression to severe complications, and a reduced mortality in COVID-19 patients treated with ACE-is (Braga et al., 2020). Cilazapril was among the CHEMBL docked compounds and was found to bind to the catalytic site of PLpro with a good stereoelectronic complementarity. Therefore, the active metabolite cilazaprilat was docked with the same docking protocol, and a docking pose similar to that of cilazapril was retrieved. Cilazaprilat makes H-bonds with the G163 backbone C=O and the G271 backbone NH, as the VIR251 cocrystallized inhibitor, and with the side chain OH of Y273 (Figure 5A). Moreover, it forms a pi-stacking interaction with H272 (docking score: −5.791 kcal/mol, MM-GBSA score: −51.74 kcal/mol, TC-IFP: 0.682).

Edoxaban and rivaroxaban are direct oral anticoagulants (DOACs) targeting factor Xa activity, commonly used in the therapy of patients with atrial fibrillation (Trujillo and Dobesh, 2014; Stacy et al., 2016). As DOACs are reported to interact with the P-glycoprotein and/or cytochrome P450-based metabolic pathways, many drugs such as antivirals administered to COVID-19 patients may interfere with their anticoagulant action. Therefore, for patients regularly assuming DOACs, clinicians have recommended to replace DOACs with heparin to avoid drug–drug interactions (Testa et al., 2020). However, in COVID-19 patients, the coagulation function is heavily unbalanced leading to hypercoagulation and the development of life-threatening coagulopathies, which may negatively affect the prognosis (Han et al., 2020; Pryzdial et al., 2020). Indeed, the alteration of blood clotting and inflammation are two frequently coupled manifestations of viral infections. Therefore, DOACs such as apixaban have shown an antiviral activity on herpes simplex virus type 1 and have been proposed as a possible therapeutic strategy to control COVID-19 (Pryzdial et al., 2020).

In our VS campaign, both rivaroxaban and edoxaban were shown to make favorable interactions within the PLpro catalytic site. As VIR251, rivaroxaban (docking score: −5.262 kcal/mol, MM-GBSA score: −68.85 kcal/mol, TC-IFP = 0.587) is hydrogen bonded to the G163 backbone C=O and the G271 backbone NH, and in addition, it forms an H-bond with the C111 NH (Figure 5C), whereas edoxaban (docking score: −7.973 kcal/mol, MM-GBSA score: −77.28 kcal/mol, TC-IFP: 0.762) is characterized by a high TC-IFP score mimicking VIR251 H-bonds with the backbone carbonyl group of G163, the backbone NH of G271, and the Y264 side chain OH. Furthermore, edoxaban is at hydrogen bond distance from the backbone C=O of Y268 and part of the compound projects toward the deep pocket flanking the S4 pocket, thus potentially exploring other still unexplored regions of the BS (Figure 5B).

#### Drugs Belonging to Other Classes

**Figure 6:**
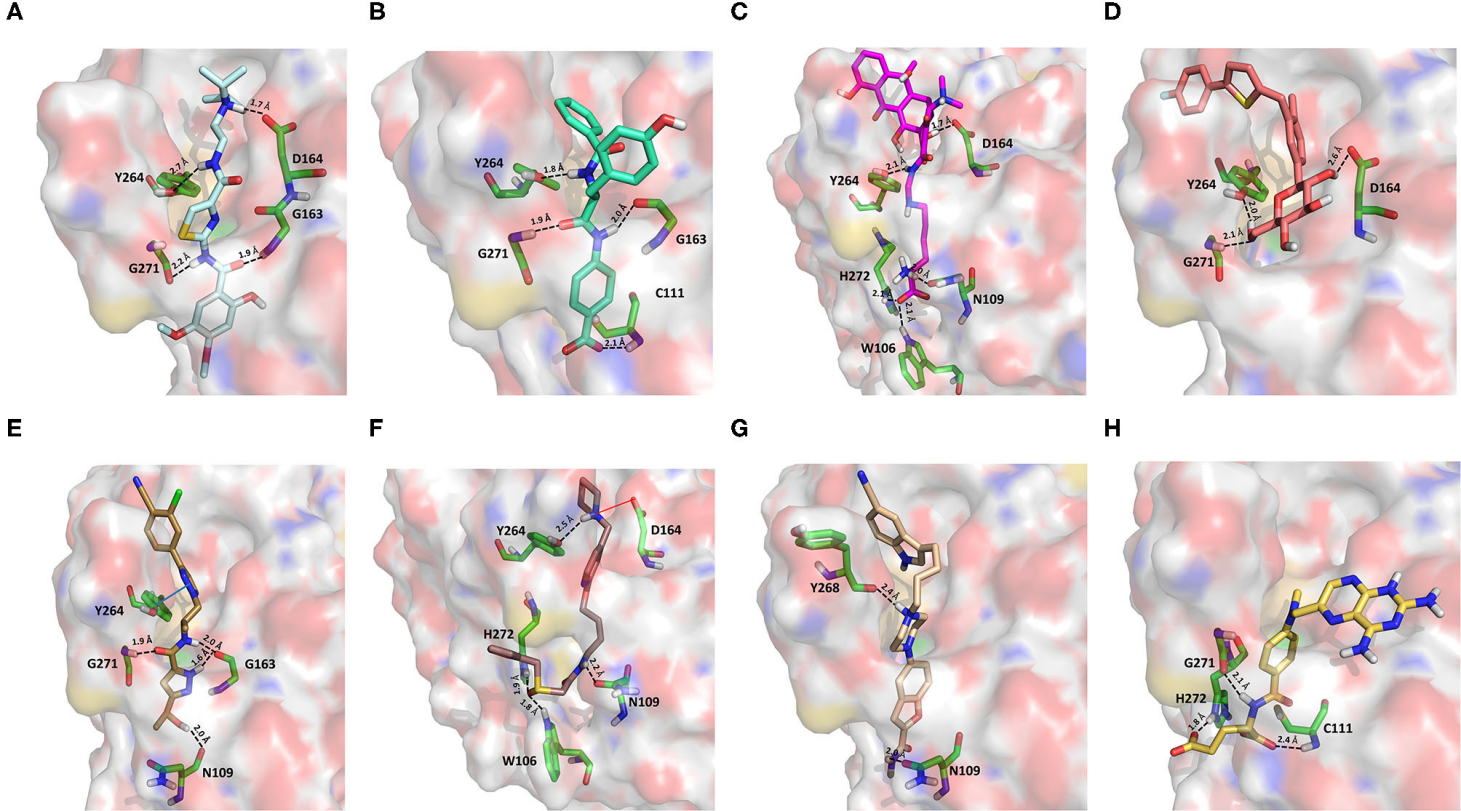
Top-scored docking poses of candidates for non-covalent PLpro inhibition belonging to different classes: **(A)** acotiamide, **(B)** bentiromide, **(C)** lymecycline, **(D)** canagliflozin, **(E)** darolutamide, **(F)** lafutidine, **(G)** viladozone, and **(H)** methotrexate. Ligands and important residues are rendered as sticks, whereas the protein is represented as a surface. H-bonds are represented by dotted black lines, whereas the salt bridge interaction between lafutidine and D164 is itemized by a red line. For the sake of clarity, only polar hydrogen atoms are shown.

Acotiamide is an acetylcholinesterase inhibitor approved in Japan for treating dyspepsia and functional dyspepsia (Bhalla, 2017). In Europe and United States, it is undergoing phase III clinical trials with promising results. Our data suggested that acotiamide may be a strong inhibitor of PLpro. As reported in Table 1, this hypothesis is supported by the computed docking (−8.07 kcal/mol) and MM-GBSA (−82.16 kcal/mol) scores, being among the best scores returned by all the screened compounds. This compound interacts with the side chains of D164 and Y264, as well as with the backbones of G163 and G271 (Figure 6A), thus reproducing the binding mode of VIR251, as also confirmed by the computed TC-IFP, being equal to 0.706.

Our data, combined with its proved high safety profile (Tack et al., 2018), make this drug an ideal candidate for further testing.

Bentiromide is an orally administrated dipeptide used in the so-called “bentiromide test” for the evaluation of the pancreatic exocrine function (Weizman et al., 1985). According to our results, bentiromide may efficiently bind to PLpro. This is mainly supported by the computed docking score (−8.80 kcal/mol), close to that returned by redocking VIR251 (−9.21 kcal/mol) and by the very high TC-IFP (0.781). Indeed, bentiromide interacts *via* H-bonds with the backbones of G163, G271, and Y264, as in the case of VIR251. In addition, an H-bond interaction with the backbone of C111 and a pi-stacking interaction with the side chain of Y264 were also observed (Figure 6B).

Lymecycline is a broad-spectrum antibiotic belonging to the tetracycline class and approved for use in Europe (Stratford, 1965). As reported in Table 1, it was predicted to have a good affinity to PLpro, with an MM-GBSA score (−94.45 kcal/mol) better than that returned by VIR251 (−91.27 kcal/mol) and a high value of TC-IFP (0.701). Lymecycline is hydrogen bonded to the side chains of D164, Y264, and W106 as VIR251. In addition, H-bond interactions with the side chains of N109 and H272 were also observed (Figure 6C). Because of their immunomodulatory and anti-inflammatory properties, combined with their well-known safety profile, tetracyclines are considered ideal candidates for repurposing against SARS-CoV-2, as recently highlighted by Singh et al. (2020). Importantly, this class of antibiotics has shown a potential efficacy in patients with ARDS, one of the most common clinical manifestations in COVID-19 patients (Singh et al., 2020).

Canagliflozin is a drug approved by the FDA for the treatment of type 2 diabetes, one of the main risk factors for severe COVID-19 outcomes (Jakher et al., 2019). In particular, it acts as an inhibitor of the sodium glucose co-transporter-2 (SGLT2). According to our data, this drug binds to PLpro with a good affinity (MM-GBSA score: −83.73 kcal/mol) and mimics the binding mode of VIR251, being able to make H-bonds with the side chains of D164 and Y264 and with the backbone of G271 (Figure 6D). Moreover, SGLT2 inhibitors have recently been proved to have a protective effect on the heart, kidney, and lung, and their potential benefits in COVID-19 patients have been hypothesized on the basis of a clinical trial showing the impact of dapagliflozin, a parent compound of canagliflozin, in patients with respiratory failure (Fernandez-Fernandez et al., 2020).

Approved by the FDA in 2019 for the treatment of metastatic castration-resistant prostate cancer (Research CDE, 2019), darolutamide is a non-steroidal antagonist of the androgen receptor. Our data indicated that this drug may efficiently bind to PLpro. Indeed, its docking pose scored better than that of VIR151 (−10.03 vs. −9.21 kcal/mol) and showed a high TC-IFP (0.698) and a good MM-GBSA score (−83.45 kcal/mol). Darolutamide makes H-bond interactions with the backbones of G163, G271, and N109, as well as a well-oriented pi-stacking interaction with Y264 (Figure 6E). As highlighted by Sugawara et al., darolutamide is responsible for a reduced expression of TMPRSS2 (Sugawara et al., 2019), the serine protease proved to be implicated in the replication of SARS-CoV and MERS-CoV infections (Montopoli et al., 2020). Notably, the TMPRSS2 involvement in SARS-CoV-2 host cell entry has recently been hypothesized on the basis of epidemiological studies indicating that the development of a serious infection is less frequent in patients treated with androgen receptor antagonists (Montopoli et al., 2020). All these evidences combined with our *in-silico* findings make darolutamide an ideal candidate for further *in vitro* testing.

Lafutidine is a new second-generation histamine H2 receptor antagonist (H2RA) with gastroprotective actions. It acts by inhibiting the daytime secretion of gastric acid by acting both directly on the H2 receptors and indirectly by increasing gastric nitric oxide production (Nakano et al., 2011). Although lafutidine has already been approved and marketed in Japan and India to treat gastric ulcers, it is still in phase III development in European Union and United States. This compound returned one of the best docking scores (−8.20 kcal/mol) among the selected molecules (Table 1) and a TC-IFP value (0.662), indicating a binding mode similar to that of the VIR251 inhibitor. As shown in Figure 6F, lafutidine was predicted to make H-bonds with the side chains of H272, W106, N109, and Y264. A T-shaped pi-stacking with Y264 and a salt bridge with D164 were also observed. It is worth to note that the administration of another H2RA (famotidine) has been associated to a reduced risk of death in COVID-19 patients (Freedberg et al., 2020). Moreover, Aguila et al. speculated that H2RAs could be a good option for COVID-19 treatment because of their ability to interfere with the gastric pH (Aguila and Cua, 2020). Therefore, its potential multitarget activity could show promise for COVID-19 treatment.

Approved by the FDA in 2011 to treat major depressive disorder, a condition that affects approximately 200 million people worldwide (Mirzaei et al., 2019), vilazodone acts as a 5-HT_1A_ receptor partial agonist and is the only drug currently defined as a serotonin partial agonist-reuptake inhibitor (SPARI) (Stahl, 2014). As reported in Table 1, it returned a promising MM-GBSA score (−70.76 kcal/mol) and a very high TC-IFP (0.742). In particular, this compound interacts with PLpro via H-bond interactions with the backbone of Y268, as VIR251, and the side chain of N109. Remarkably, the orientation of its indole substituent within the S4 cavity is almost superimposable with the crystallographic coordinates of VIR251, as shown in Figure 6G. If confirmed by experiments, vilazodone ability to inhibit PLpro would be particularly appealing for the treatment of COVID-19 patients with neurological manifestations (Armocida et al., 2020), because of its ability to efficiently cross the blood–brain barrier (Bundgaard et al., 2016).

Methotrexate is a well-known antineoplastic, immunosuppressive, and anti-inflammatory agent that inhibits dihydrofolate reductase preventing the formation of tetrahydrofolate, which is required for DNA synthesis (Hannoodee and Mittal, 2020). Because of its anti-inflammatory effects, it has been recently proposed for the treatment of COVID-19 hyperinflammation (Safavi and Nath, 2020). In our study, we found that methotrexate (docking score: −5.417 kcal/mol, MM-GBSA score: −38.42 kcal/mol, TC-IFP: 0.458) is able to bind to the PLpro catalytic site, albeit exploring a partially different hydrogen bond network compared to VIR251. Indeed, methotrexate makes hydrogen bond interactions with the G271 backbone C=O, the backbone NH of C111, and the side chains of C111 and H272 (Figure 6H).

### Candidates for Covalent PLpro Inhibition

All the compounds belonging to the developed dataset and including Michael acceptor (MA) groups, potentially able to alkylate C111 as the VIR251 cocrystallized inhibitor, were submitted to covalent docking simulations. As reviewed some years ago by Santos et al., MA groups can be responsible for an irreversible and very effective inactivation of cysteine proteases (Santos and Moreira, 2007). A covalent mechanism of action, indeed, leads to several advantages in terms of potency, duration of action, and selectivity (Singh et al., 2011) and therefore is highly desirable for COVID-19 treatment. On the basis of the obtained docking scores and visual inspection, two compounds were selected as the best candidates for a covalent inhibition of PLpro (Table 2).

**Table 2:** Candidate drugs for covalent PLpro inhibition.

**CHEMBL ID**	**Compound**	**2D structure**	**Docking score (rank)**	**TC-IFP (rank)**
**/**	**VIR251**		**−10.08**	**—**
116438	Curcumin	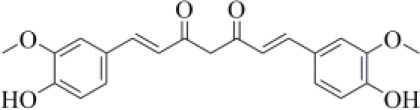	−8.05 (4)	0.609 (7)
1173655	Afatinib	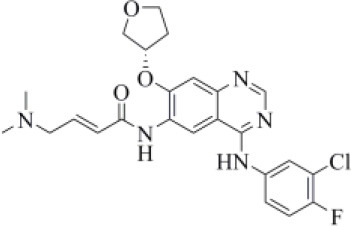	−5.80 (10)	0.603 (9)

*Docking scores are reported in kcal/mol. The numbers in brackets indicate the corresponding rank positions. The docking score obtained by redocking VIR251 (covalent docking protocol) is also reported*.

**Figure 7:**
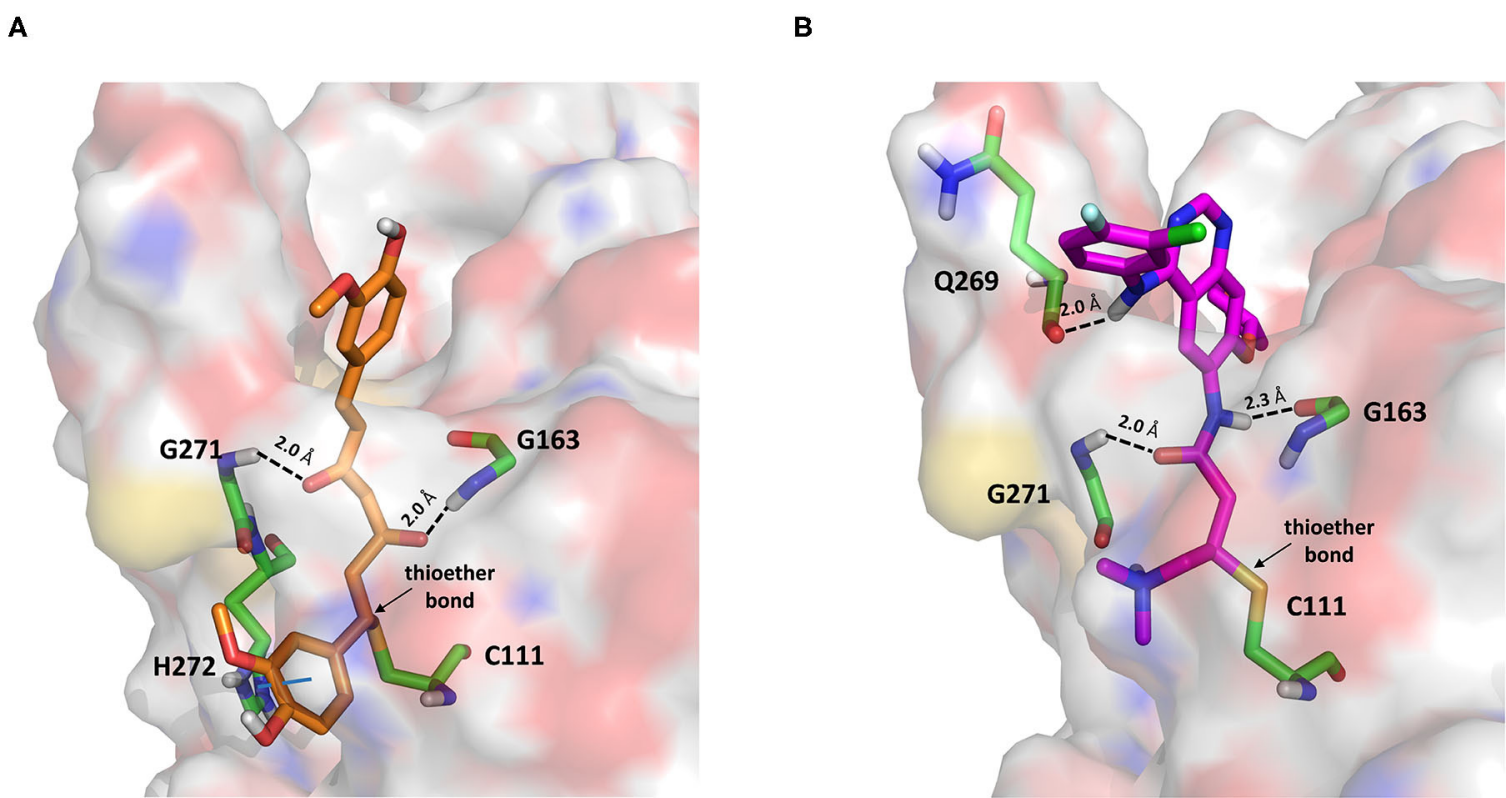
Top-scored covalent docking poses of **(A)** curcumin and **(B)** afatinib. Ligands and important residues are rendered as sticks, whereas the protein is represented as a surface. H-bonds are represented by dotted black lines, whereas the pi-stacking interaction between curcumin and H272 is itemized by a blue line. For the sake of clarity, only polar hydrogen atoms are shown.

Curcumin is a polyphenol extracted from an East Indian plant *Curcuma longa* that reached a phase III clinical trial for the treatment of inoperable pancreatic cancer (Hatcher et al., 2008). It has a long history of use as a food additive due to its potent anti-inflammatory and antioxidative properties (Praditya et al., 2019), whereas no toxicity concerns are associated with its administration. This compound returned one of the best docking scores among all the screened molecules (−8.051 kcal/mol) and its top-scored docking pose mimics some key interactions observed for VIR251 such as those with the backbones of G163 and G271 (TC-IFP: 0.609). Finally, a pi-stacking with H272 was also observed (Figure 7A).

Importantly, the ability of curcumin to interact, *via* a Michael addition, with a cysteine residue has been well-documented by studies on other pharmacological targets such as the myeloid differentiation protein 2 (Gradišar et al., 2007) and the transcription factor STAT-3 (Hahn et al., 2018). Furthermore, the antiviral properties of this compound against several viruses have recently been reviewed by Praditya et al. (2019). In particular, curcumin exhibited a significant inhibitory effect against SARS-CoV (Wen et al., 2007). Last but not least, a recent study has confirmed that this polyphenol exerts a protective effect on the lung in case of severe pneumonia caused by SARS-CoV-2, decreasing the expression of proinflammatory cytokines (IL-6, IL-8, and IL-10) (Liu and Ying, 2020). All these evidences, combined with our findings, make curcumin an ideal candidate for further investigations.

Afatinib is a tyrosine kinase inhibitor approved by the FDA in 2013 for the treatment of advanced non–small cell lung cancer (Deeks and Keating, 2018). Interestingly, it is a well-known covalent inhibitor of different proteins belonging to the ErbB family such as, e.g., the epidermal growth factor receptor (Deeks and Keating, 2018). Indeed, the presence of an MA group allows the reaction with a conserved cysteine residue of the catalytic cleft (Yu et al., 2018). According to the obtained docking score (−5.799 kcal/mol) and pose, herein we hypothesize that afatinib could bind to PLpro by means of the same mechanism observed in different tyrosine kinases. Afatinib is well-accommodated in the catalytic site (TC-IFP: 0.603) making H-bond interactions with the backbone of G171, G163, and Q269 (Figure 7B). Noteworthy, ErbB receptors have recently been hypothesized to have an important role in different stages of viral infections (Ho et al., 2017), such as host cell entry and proliferation; hence, afatinib can be considered as a drug with the potential of targeting both host proteins engaged by the virus and viral targets.

## Conclusions

In this article, we selected 24 known drugs as promising non-covalent (22) and covalent (2) inhibitors of the SARS-CoV-2 papain-like protease for the treatment of COVID-19 patients. All the compounds were selected through a structure-based computational screening performed by using, for the first time, the crystal structure of PLpro in complex with an inhibitor. This study differs from other *in silico* screenings performed for repurposing drugs on SARS-CoV-2 protein targets as we (i) extended our investigation to compounds that have reached phase III clinical trial; (ii) rescored the obtained docking poses on the basis of their computed IFPs; and (iii) performed both non-covalent and covalent docking simulations. The selected compounds, belonging to different pharmacological classes, such as that of protease inhibitors (amprenavir, indinavir, anagliptin, boceprevir, and semagacestat), adrenergic receptor modulators (vilanterol, arformeterol, atenolol), anticoagulants (edoxaban and rivaroxaban), ACE-is (cilazapril), antidiabetes (anagliptin, canagliflozin), PK inhibitors (dasatinib, pexidartinib, copanlisib, and afatinib), and antiandrogens (darolutamide), can be considered as promising candidates for further *in vitro* testing to select or discard them as SARS-CoV-2 papain-like protease inhibitors. Importantly, according to the available literature data and well-reported clinical trials, all the proposed compounds have a known safety profile, and for the majority of them, polyphamacological effects highly desirable to treat COVID-19 patients can be hypothesized because of the concomitant inhibition of viral and host proteins involved in viral infection. Therefore, once their antiviral activity could be confirmed, these drugs may represent a ready-to-use treatment for hindering SARS-CoV-2 devastating effects.

## Data Availability Statement

The raw data supporting the conclusions of this article will be made available by the authors, without undue reservation.

## Author Contributions

PD performed the calculations, supervised by FC and GM. All authors contributed to the design and implementation of the research, to the analysis of the results and to the writing of the manuscript.

## Conflict of Interest

The authors declare that the research was conducted in the absence of any commercial or financial relationships that could be construed as a potential conflict of interest.
